# Editorial

**DOI:** 10.1051/parasite/2012194295

**Published:** 2012-11-15

**Authors:** René Houin

Twenty years ago, the French Parasitology Society bought out its publisher, thanks to the profit made during the ICOPA VII. The journal *Annales de Parasitologie humaine et comparée*, which had been created in 1923 by Emile Brumpt with the help of Maurice Langeron and Maurice Neveu-Lemaire, became *Parasite*, a journal dedicated to continuing scientific dissemination of French parasitologists. The *Annales de Parasitologie humaine et comparée*, themselves succeded to the *Archives de Parasitologie* created by Raphaël Blanchard in the previous century. In the first issue of *Parasite*, the President of the French society of Parasitology, Jean Antoine Rioux, stressed the impact of new scientific approaches, but also on the need for the integration of various currents of thought. At the same time, Wallace Peters, member of the Scientific Board of the new journal paid a warm tribute to Alain Gabriel Chabaud, who had edited the *Annales de Parasitologie humaine et comparée* for the previous twenty years.

Keeping a parasitology journal alive until today is the result of strong and skillful team-work. This united team included the board of French Society of Parasitology, and in addition a Scientific Board made up of prominent French and foreign parasitologists, and a high level Editorial Board. Over the years, these different boards were supplemented with new competences when necessary. Their effectiveness has guaranteed the quality of published manuscripts and now *Parasite* has an international standing amongst the journals indexed in parasitology. The role of the Editor-in-Chief, Jean-Marc Dumas has been also decisive, due to him being a parasitologist with training in scientific publishing (he had notably been Editor-in-Chief of the *Journal du CNRS* for five years). Together with his wife Gisèle Gilkes-Dumas, he had just created the Princeps publishing company that provided *Parasite* with logistics and rigorous management without which such an undertaking would not have occurred. Thanks to its wide range of topics, *Parasite* was and is able to disseminate the work of parasitologists worldwide: this editorial is an opportunity to thank all the contributors and to encourage them to continue this partnership.

The journal *Parasite* changes, but continues to serve the parasitology research community! Scientific publishing is currently experiencing the great upheaval of the online publication and free access. Over the last two years *Parasite* has followed this trend without abandoning the paper edition. Adapting to these new conditions of publication justifies both a change of publisher and editorial team. However, the spirit of the journal continues and its fascination for “*this object of natural history that is a parasite*” described so eloquently by Jean Antoine Rioux’s editorial twenty years ago. In closing, I wish a great success to the new editorial team of *Parasite*.

